# Exploring community rates of food assistance and diabetes among older adults in MA, RI, and CT

**DOI:** 10.1093/geroni/igaf122.2432

**Published:** 2025-12-31

**Authors:** Calvin Tran, Qinglin Gao, Mengshi Liu, Nachalie Rodriguez-Cruz, Elizabeth Dugan

**Affiliations:** University of Massachusetts Boston, Boston, Massachusetts, United States; University of Massachusetts Boston, Boston, Massachusetts, United States; University of Massachusetts Boston, Boston, Massachusetts, United States; University of Massachusetts Boston, Boston, Massachusetts, United States; University of Massachusetts Boston, Boston, Massachusetts, United States

## Abstract

Food insecurity and poor nutritional intake has serious health consequences. Poor nutrition has been associated with diabetes and eating healthy is often recommended to manage blood sugar. This study aims to examine the association at the population level to explore if community rates of obtaining food benefits (SNAP) are associated with diabetes rates in adults 65+. Understanding community rates of food stamp participation can identify barriers to food access and can inform programs and interventions to address diabetes among older adults across MA, RI, and CT. The current study uses results from the 2025 Healthy Aging Data Report (healthyagingdatareports.org). Indicators were calculated data from the American Community Survey (2018-2022 MA, RI, CT) and the Centers for Medicare and Medicaid Services Beneficiary Summary File (2020-2021 MA, RI, CT). Small area estimation techniques were used to calculate age- and sex-adjusted rates, and ArcGIS software was used for mapping and geographic analyses. Results showed that state average rates for food benefits were MA 13.34% (0-48.87%), RI 14.73% (2.18-33.98%), and CT 11.04% (0-42.62%). Rhode Island had the highest average state rate but the smallest range in rates. Rural communities had the lowest rates of food stamp participation. Diabetes: MA 28.62% (15.00-48.98%), RI 32.43% (15.80-33.98%), and CT 31.78% (18.13-46.89%). Other environmental risk factors such as lack of infrastructure for physical activity or food apartheids may contribute to some of the differences observed. Understanding the difference in community rates across New England can better inform policymakers in addressing diabetes maintenance and prevention among older adults.

